# Towards a Customized Oral Drug Therapy for Pediatric Applications: Chewable Propranolol Gel Tablets Printed by an Automated Extrusion-Based Material Deposition Method

**DOI:** 10.3390/pharmaceutics17070881

**Published:** 2025-07-04

**Authors:** Kristiine Roostar, Andres Meos, Ivo Laidmäe, Jaan Aruväli, Heikki Räikkönen, Leena Peltonen, Sari Airaksinen, Niklas Sandler Topelius, Jyrki Heinämäki, Urve Paaver

**Affiliations:** 1 Institute of Pharmacy, Faculty of Medicine, University of Tartu, Nooruse 1, 50411 Tartu, Estonia; 2 Department of Geology, Institute of Ecology and Earth Sciences, University of Tartu, Ravila 14a, 50411 Tartu, Estonia; 3 Division of Pharmaceutical Chemistry and Technology, Faculty of Pharmacy, University of Helsinki, Viikinkaari 5E, 00014 Helsinki, Finland; 4 CurifyLabs Oy, Salmisaarenaukio 1, 00180 Helsinki, Finland

**Keywords:** automated semi-solid extrusion (SSE) material deposition, formulation development, chewable gel tablets, propranolol hydrochloride, mechanical properties, drug release in vitro

## Abstract

**Background:** Automated semi-solid extrusion (SSE) material deposition is a promising new technology for preparing personalized medicines for different patient groups and veterinary applications. The technology enables the preparation of custom-made oral elastic gel tablets of active pharmaceutical ingredient (API) by using a semi-solid polymeric printing ink. **Methods**: An automated SSE material deposition method was used for generating chewable gel tablets loaded with propranolol hydrochloride (-HCl) at three different API content levels (3.0 mg, 4.0 mg, 5.0 mg). The physical appearance, surface morphology, dimensions, mass and mass variation, process-derived solid-state changes, mechanical properties, and in-vitro drug release of the gel tablets were studied. **Results**: The inclusion of API (1% *w*/*w*) in the semi-solid CuraBlend^TM^ printing mixture decreased viscosity and increased fluidity, thus promoting the spreading of the mixture on the printed (material deposition) bed and the printing performance of the gel tablets. The printed gel tablets were elastic, soft, jelly-like, chewable preparations. The mechanical properties of the gel tablets were dependent on the printing ink composition (i.e., with or without propranolol HCl). The maximum load for the final deformation of the CuraBlend™-API (3.0 mg) gel tablets was very uniform, ranging from 73 N to 80 N. The in-vitro dissolution test showed that more than 85% of the drug load was released within 15–20 min, thus verifying the immediate-release behavior of these drug preparations. **Conclusions**: Automated SSE material deposition as a modified 3D printing method is a feasible technology for preparing customized oral chewable gel tablets of propranolol HCl.

## 1. Introduction

**Figure 1:**
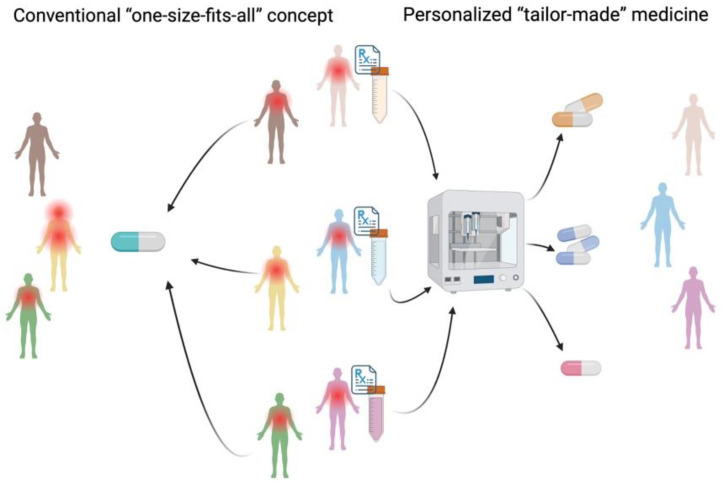
Comparison of a conventional “one-size-fits-all” concept and personalized “tailor-made” concept using pharmaceutical 3D printing. Created with BioRender.com.

Today, three-dimensional (3D) printing technologies hold great promise in preparing customized (“tailor-made”) medicines and personalized drug delivery systems (DDS) to improve the patient experience and clinical outcomes [1,2,3]. It is well known that conventional manufacturing and a “one-size-fits-all” concept are not suitable for all patient groups and for all medical conditions or diseases [4,5]. Pharmaceutical 3D printing could enable the production of small batches of products on demand, which could improve the efficiency and flexibility of hospitals and pharmacies [6]. Therefore, pharmaceutical 3D printing technologies are expected to make a revolutionary contribution to the new approach to personalized medicine. In personalized drug therapy and treatment, each patient is given a medicine that meets their needs in a suitable dosage, composition, and dosage form (Figure 1).

Oral pharmaceutical solid dosage forms (i.e., tablets, capsules, and granules) are widely used due to their simplicity and low invasiveness in both human and veterinary drug treatments. Traditionally, oral pharmaceutical dosage forms are manufactured batchwise on an industrial scale using various well-known technologies and a relatively large number of excipients. The manufacturing process of solid dosage forms, such as tablets, can be very complex, involving series of sequential steps (granulating, drying, mixing, tableting, coating), while 3D printing enables different steps to be merged for certain active pharmaceutical ingredient (API) and printing ink combinations [7]. Within the last 10–15 years, a number of promising 3D printing technologies have been introduced for pharmaceutical applications. A revolution in and the widespread use of 3D printing technology is expected [8].

The automated semi-solid extrusion (SSE) material deposition method is a new, modified form of 3D printing technology for pharmaceutical applications. Automated SSE material deposition, like traditional 3D printing methods, enables significant flexibility in designing and preparing oral solid dosage forms, making it a suitable technology for preparing personalized dosage forms for pharmaceutical applications [9]. Automated SSE material deposition is the method of choice for pharmaceutical polymer-based printing and dose dispensing due to its simplicity, precision, efficiency, low operating temperature, and suitability for preparing even high drug-loaded preparations [10,11]. The advantages of automated SSE material deposition include accessibility in the preparation of customized medications and standardized workflows [11]. Recently, automated SSE material deposition was successfully used in the preparation of customized immediate-release clopidogrel tablets intended for pediatric applications [12]. The technology also provides an opportunity to design and generate oral chewable dosage forms. Chewable tablets are administered without water, thus increasing patient compliance. Moreover, chewable tablets are not constrained by size, as they are designed to be chewed before they are swallowed [13].

**Figure 2:**
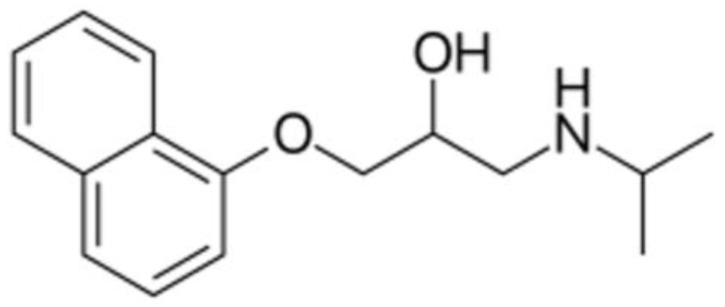
Chemical structure of propranolol.

Propranolol (Figure 2) is a non-selective beta blocker used in the treatment of common diseases such as high blood pressure and heart arrhythmia, and less often for the treatment of performance anxiety and essential tremors. Furthermore, propranolol is considered as a feasible model API for the development of pediatric formulations because it is chemically stable and analytically detectable at low concentrations. Since children are not just “small adults”, personalized drug therapy and treatment are needed with pediatric patients. For example, the initial daily dose of propranolol for infants depends on the treatment, ranging from 1 mg/kg to 1.5 mg/kg [14]. Therefore, individualized dosing of propranolol for special patient groups is of the utmost importance, and this process should include improving organoleptic properties. The pediatric need for immediate-release low-dose propranolol formulations makes this API an ideal candidate for use as an example to be formulated in orodispersible DDSs [15].

To date, only a few research works have been published on the pharmaceutical 3D printing of propranolol drug preparations. Zhu and colleagues [16] developed 3D-printed gummy chewable propranolol tablets for pediatric administration using a printing ink containing gelatin and carrageenan in extrusion-based printing. The binary semi-solid printing mixtures of gelatin and carrageenan were found to improve the thermal stability, printability, administration, and drug release properties of the printed gummy tablets. Jovanovic et al. [17] used SSE 3D printing to prepare gelatin-based mucoadhesive buccal films loaded with propranolol HCl for pediatric and geriatric administration. The inclusion of synthetic polymers such as povidone and polyvinyl alcohol (PVA) in the gelatin-based printing ink enhanced the printing performance, mechanical and mucoadhesive properties, and prolonged drug release behavior of the buccal films. Such 3D-printed oral DDS developed for propranolol hydrochloride successfully bypassed the first-pass metabolism and provided the prolonged effects of API with reduced dose variability. More recently, Alqahtani et al. [18] introduced a gastric floating tablet with prolonged gastric floating time and a sustained drug release profile printed using a 3D printing technique based on fused deposition modelling. In summary, the abovementioned 3D-printed drug preparations (chewable tablets, semi-solid buccal films, and a gastro-retentive floating system) enabled drug release to be controlled and therapeutic effects to be extended, thus improving oral bioavailability and patient compliance. Such personalized drug carriers offer effective solutions to pharmaceutical challenges such as the poor bioavailability of API and inadequate patient adherence, and could therefore significantly extend possibilities for individualized treatments.

The aim of this study was to develop automated SSE material deposition technology for preparing novel customized chewable gel tablets intended for pediatric applications, and to investigate the printing performance and final properties of such tablets. The chewable gel tablets loaded with propranolol hydrochloride (-HCl) at three different target content levels (3.0 mg, 4.0 mg, and 5.0 mg) were generated using a commercial Cura-Blend™ polymeric printing gel. The effects of API on the viscosity of the printing gel and the printing performance of the gel tablets were investigated. The physical appearance, dimensions, mass and mass variation, solid-state properties, mechanical properties, and in-vitro drug release of the gel tablets were studied. To date, the properties of individualized gel tablets manufactured using comparable techniques have not been systematically investigated. The findings of this study contribute novel insights into the formulation and characterization of such printed dosage forms and highlight the potential of this approach for the future development of patient-tailored medicines intended for the clinical use.

## 2. Materials and Methods

### 2.1. Materials

CuraBlend™ (CurifyLabs Oy, Helsinki, Finland) was used as a polymeric printing gel within the automated SSE material deposition method. CuraBlend^TM^ is a multicomponent mixture consisting of gelatin, cocoa butter, and purified water, as well as small amounts of additives supporting the SSE 3D-printing. The pre-mixture of CuraBlend^TM^ was prepared in CurifyLabs Oy (batch B10623, prepared on 2 May 2023), and it was used within one week for SSE 3D-printing.

Propranolol HCl (Thermo Scientific, Tokyo, Japan) powder (purity 99%) was used as the API in the chewable gel tablets. Hydrochloric acid (≥37%, Sigma-Aldrich, Darmstadt, Germany) and purified water (Millipore Milli-Ro 12 Plus, Merck KgaA, Darmstadt, Germany) were used for preparing the dissolution medium. All reagents used in the HPLC analysis were of European Pharmacopoeia (Ph.Eur.) quality and ordered from Sigma-Aldrich (Germany).

### 2.2. Methods

#### 2.2.1. Viscosity Measurements of Semi-Solid Printing Mixtures

The viscosity of the semi-solid mixtures (printing gels) was studied at 45 ± 5 °C with a Brookfield Ametek DVNext viscosimeter (Brookfield Engineering, Middleboro, MA, USA). The measurements were performed in triplicate with the reference CuraBlend™ semi-solid printing mixture (without API) and CuraBlend™ semi-solid printing mixture loaded with propranolol HCl (1% *w*/*w*). The measurements were performed using a constant rotation speed of 0.5 rpm, a shear rate of 1.000, and torque of 18–18.9%.

#### 2.2.2. Automated Semi-Solid Extrusion (SSE) Material Deposition

The automated MiniLab SSE material deposition system (Natural Machines, Barcelona, Spain) was used to prepare the chewable gel tablets. Several printing cycles were performed. The automated SSE material deposition process took place at 42 °C and at ambient relative humidity (RH 32%).

**Figure 3:**
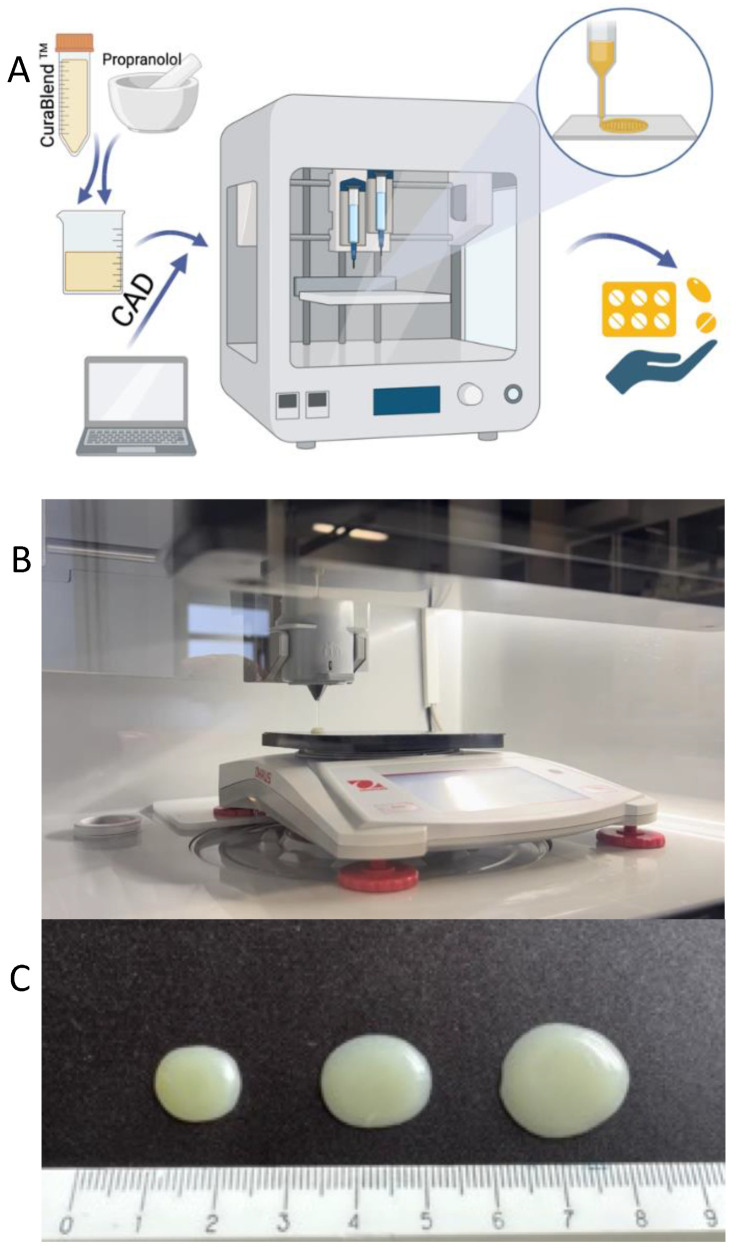
(**A**) Schematic illustration of the automated semi-solid extrusion (SSE) material deposition process. Key: CAD = computer-aided design; (**B**) Photograph of the automated SSE material deposition system and pre-filled syringe based on the predetermined amount or weight of material; (**C**) A representative photograph of the gel tablets prepared using the automated SSE material deposition method. The gel tablets are loaded with 1% (*w*/*w*) propranolol HCl. From left to right, the target weight of the gel tablets is 300.0 mg, 400.0 mg, and 500.0 mg.

A schematic illustration of the automated SSE material deposition process is given in Figure 3A. For preparing the reference gel tablets (without API), the printing mixture was first heated to a temperature of 42–45 °C in a water bath. When the mixture reached the predetermined temperature and a semi-solid state with a uniform flow, it was transferred to the printing syringe. The syringe was closed and placed in the preheated syringe slot of the MiniLab 3D SSE material deposition system. The syringe was kept in the system for 15 min to ensure that the printing material was at a uniform temperature throughout the syringe. The number of gel tablets and the amount of API (determined based on the weight of the tablets) were entered as printing parameters into the printer management program (Printer Web App, CurifyLabs Oy, Helsinki, Finland). At the beginning of every SSE material deposition process, checks were made to ensure that the printing mixture exited the syringe freely and that there was no blockage or other obstruction at the opening of the syringe. Figure 3B shows the automated SSE material deposition system in operation, while Figure 3C shows the representative gel tablets loaded with 1% (*w*/*w*) propranolol HCl.

For preparing the API-loaded gel tablets, the printing mixture was heated to a temperature of 42–45 °C in a water bath. The required amount of propranolol HCl was weighed, taking into account that the target concentration of API in the gel tablets is 1% (*w*/*w*), and the content of API was adjusted based on the tablet weight. When the semi-solid printing mixture reached the predetermined temperature (42–45 °C), the required amount of API was weighed and added to the mixture, and finally mixed until the mixture was homogeneous. The mixture was then transferred to a printing syringe, and the process continued as described previously with the reference gel tablets.

A total of eight to nine reference gel tablets (without an API load) were printed for the control preparations of the contents of each gel tablet (3.0 mg, 4.0 mg, and 5.0 mg), with the target weight of these reference gel tablets being 300.0 mg, 400.0 mg, and 500.0 mg, respectively. A total of 75 gel tablets with theoretical API content of 3.0 mg (*n* = 25), 4.0 mg (*n* = 25) or 5.0 mg (*n* = 25) were prepared, and the target weight of these drug-loaded gel tablets was 300.0 mg, 400.0 mg, and 500.0 mg, respectively. Figure 3C shows the representative gel tablets for each gel tablet size. For a short-term stability study, we printed an additional twelve (12) gel tablets with an API load of 3.0 mg and 5.0 mg.

After printing, the gel tablets were placed in a refrigerator (+2–8 °C) to solidify for 30 min. The physical appearance, mass and mass variation, and dimensions (length, weight, and height) of the gel tablets were studied after the solidification period. After the measurements, the gel tablets were carefully packed in a blister pack and stored in the refrigerator (+2–8 °C) for future studies.

#### 2.2.3. Physical Appearance, Mass, and Dimensions of Gel Tablets

The physical appearance (i.e., layout, shape, and potential printing defects) of the gel tablets was visually inspected. The average weight and weight variation of the gel tablets were determined using an analytical balance Denver Instrument APX-200 (Cole-Parmer Instrument Company LLC, Vernon Hills, IL, USA), and the dimensions (length, width, height) were measured with a digital micrometer Ironside 150 mm (Ironside International, Paris, France). Measuring the height of the gel tablets was somewhat challenging, since the freshly printed gel tablets were soft and delicate. Therefore, the measurement of height needed to be conducted with care, i.e., without accidentally compressing the surface of the gel tablet.

#### 2.2.4. Scanning Electron Microscopy

The surface structure and morphology of the gel tablets were investigated using a high-resolution scanning electron microscope (SEM; Zeiss EVO^®^ 15 MA, Oberkochen, Germany). The samples were mounted on aluminum stubs with a conductive carbon film, and were magnetron-sputter coated with a 3-nm platinum layer in an argon atmosphere before microscopy.

#### 2.2.5. Fourier Transform-Infrared Spectroscopy

The physical solid-state properties of the gel tablets were investigated with Fourier transform-infrared (FTIR) spectroscopy using a Shimadzu IRPrestige-21 spectrometer (Shimadzu Europa GmbH, Duisburg, Germany) with Specac Golden Gate ATR (Specac Ltd., Orpington, UK). The FTIR spectra of pure propranolol HCl powder, cocoa butter, xylitol, and CuraBlend™ printing mixture without or with API (1% *w*/*w*), were used as the references for the FTIR spectra of the printed gel tablets. The samples were scanned over a range of 4000–600 cm^−1^ at a resolution of 4 cm^−1^ for 20 scans on the three different spots.

#### 2.2.6. X-Ray Powder Diffraction

The X-ray powder diffraction (XRPD) patterns of the raw materials and 3D-printed gel tablets were obtained using an X-ray diffractometer (D8 Advance, Bruker AXS GmbH, Karlsruhe, Germany). The XRPD experiments were carried out in symmetrical reflection mode (Bragg–Brentano geometry) with CuKα radiation (1.54 Å). The angular range was from 5° 2-theta to 35° 2-theta with steps of 0.2° 2-theta. The scattered intensities were measured with a LynxEye 1-dimensional detector with 165 channels. The operating voltage and current were 40 kV and 40 mA, respectively.

#### 2.2.7. Mechanical Tests

**Figure 4:**
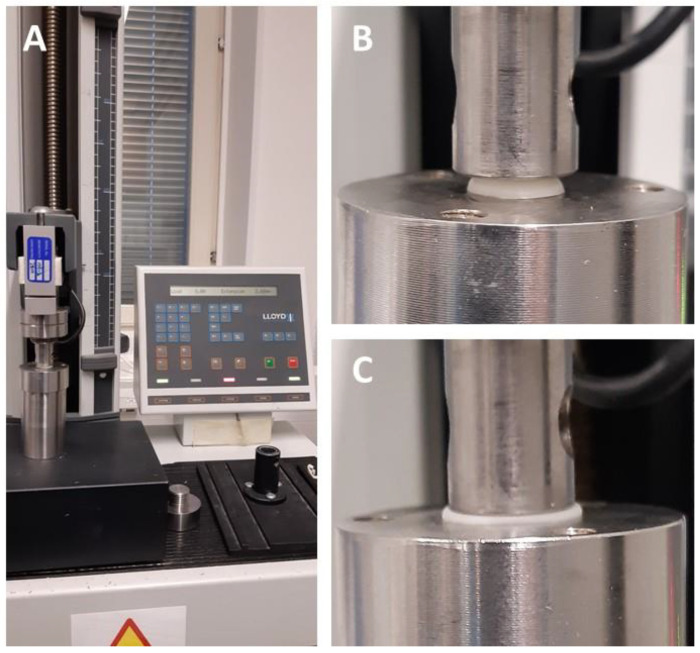
(**A**) A Lloyd LRX material tester equipped with Nexygen software for force-time measurement and data analysis. The representative photographs in (**B**,**C**) depict the start and end-phase, respectively, of the mechanical test of CuraBlend™ gel tablets (*n* = 6) in a Lloyd LRX tester.

The mechanical properties of the CuraBlend™ gel tablets were studied with a Lloyd LRX material testing device (Lloyd LRX, Lloyd Ltd., London, UK) equipped with NexygTM MT Materials Test and Data Analysis Software and OndioTM Application Builder Software packages for measuring and data analysis (Figure 4A). Figure 4B,C presents representative photographs of the starting point and end-phase of the mechanical test performed for the CuraBlend™ gel tablets, respectively. The constant speed of the crosshead in the tests was 1.000 mm/min. The gauge length used in the measurements was 5.00 mm, and the area was 3.00 × 4.00 mm. The test was automatically terminated at a load of 100 N. A total of six (6) parallel gel tablets were measured. All mechanical tests were conducted at ambient room temperature and in conditions of relative humidity (22 ± 2 °C).

#### 2.2.8. Dissolution Test In Vitro

The in-vitro dissolution of the gel tablets was investigated using a Sotax AT 7 Smart dissolution test apparatus (Sotax AG, Aesch, Switzerland) equipped with an Ismatec IPC 8 ISM 931 peristaltic pump (Cole-Parmer Instrument Company LLC, Vernon Hills, IL, USA) and a Specord 200 Plus spectrophotometer (Analytik Jena GmbH, Jena, Germany). A comparison of the dissolution test method described in the United States Pharmacopoiea (USP 35) for Propranolol Hydrochloride Tablets and our modified in-house dissolution test method is shown in Table 1. The dissolution medium was 900 mL of 0.063 M hydrochloric acid, HCl (36.7 ± 0.2 °C), and the rotating rate of both paddles and baskets was 100 rpm. The parallel number of samples used in each dissolution test was 4–6. In the dissolution tests of the gel tablets, samples were taken at regular intervals (5 min, 10 min, 20 min, 30 min). In the additional dissolution test of the gel tablets with a drug load of 5.0 mg, the samples were taken at 1 min, 2 min, 4 min, 8 min, 16 min, and 32 min, using the method described previously. For sampling, 1.0 mL of solution was pipetted into Eppendorf tubes, and the tubes were subsequently centrifuged at 15,000× *g* for 5 min. Then, 0.8 mL of the solution was pipetted into glass vials, and the vials were placed in a refrigerator (+2–8 °C).

**Figure 5:**
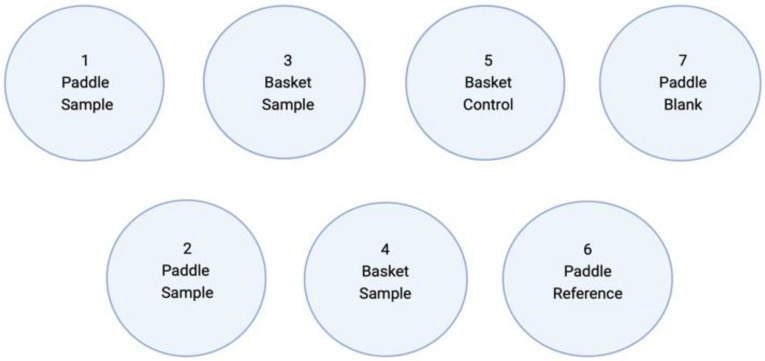
Schematic illustration of the dissolution test protocol of the gel tablets prepared using the automated semi-solid extrusion (SSE) material deposition method.

Figure 5 is a schematic illustration of the dissolution test protocol used in this study. Since there is no official or specific dissolution testing protocol for 3D-printed or automated SSE material-deposited gel tablets, we used both the paddle and basket methods in our in-house dissolution test (modified from the USP 35 dissolution test method).

#### 2.2.9. High-Performance Liquid Chromatography

The in-vitro drug release of the gel tablets were determined by means of high-performance liquid chromatography (HPLC). The HPLC system consisted of a Shimadzu HPLC Prominence modular system (Shimadzu Europa GmbH, Duisburg, Germany), which was composed of two LC-20AD high-pressure pumps, a Nexera X2 SIL-30AC autosampler, a SPD-M20A detector, and a CTO-20AC column oven. A SphereClone (TM) 250 × 4.6 mm column (Phenomenex, Torrance, CA, USA) was used.

A modified Ph. Eur. HPLC method was used for the quantitative analysis of propranolol HCl (Ph.Eur. 11.2, 2023). The stationary phase sorbent was octadecylsilyl silica gel. The mobile phase eluent consisted of tetrabutylammonium dihydrogen phosphate R, sodium lauryl sulfate R, sulfuric acid, water for chromatography, and acetonitrile. The pH of the eluent was adjusted to 3.3 with dilute sodium hydroxide solution R. The flow rate was 1.0 mL/min (which was modified since, according to Ph. Eur., the flow rate should be 1.8 mL/min). The amount of injected sample was 20 µL, and the analysis was performed at a temperature of 30 °C. An analytical wavelength of 292 nm was used on the spectrophotometric detector.

#### 2.2.10. Statistical Analysis

Microsoft Excel 2021 Pro was used for data processing, statistical calculations, graphs, and figures. The chemical structure of propranolol (Figure 2) was created with Chemaxon Marvin. BioRender.com was used to generate Figure 1 and Figure 3A. CurifyLabs Minilabs control software was used to control the printer, and a WinTDS control program was used for the dissolution test.

## 3. Results and Discussion

### 3.1. Viscosity of Semi-Solid Printing Mixtures

The viscosity of the semi-solid printing mixture loaded with propranolol HCl (1% *w*/*w*) was 2623 ± 860 cP (*n* = 3) at 42 °C, while the viscosity of the reference printing mixture (without API) was 3485 ± 199 cP (*n* = 3). The inclusion of API (1% *w*/*w*) in the printing mixture decreased viscosity and increased fluidity, thus enhancing the spread of the mixture on the printed (material deposition) bed. Due to the higher fluidity, the printing mixture tended to come out of the printing syringe, which resulted in changes in the mass and dimensions of the gel tablets. We found that, if the printing mixture was kept in the water bath during preheating for too long a period, or if a slightly higher temperature was used, the viscosity (and/or density) of the printing mixture changed. This in turn changed the mass of the mixture, which was expected to flow out during automated SSE material deposition.

### 3.2. Physical Appearance of 3D-Printed Gel Tablets

**Figure 6:**
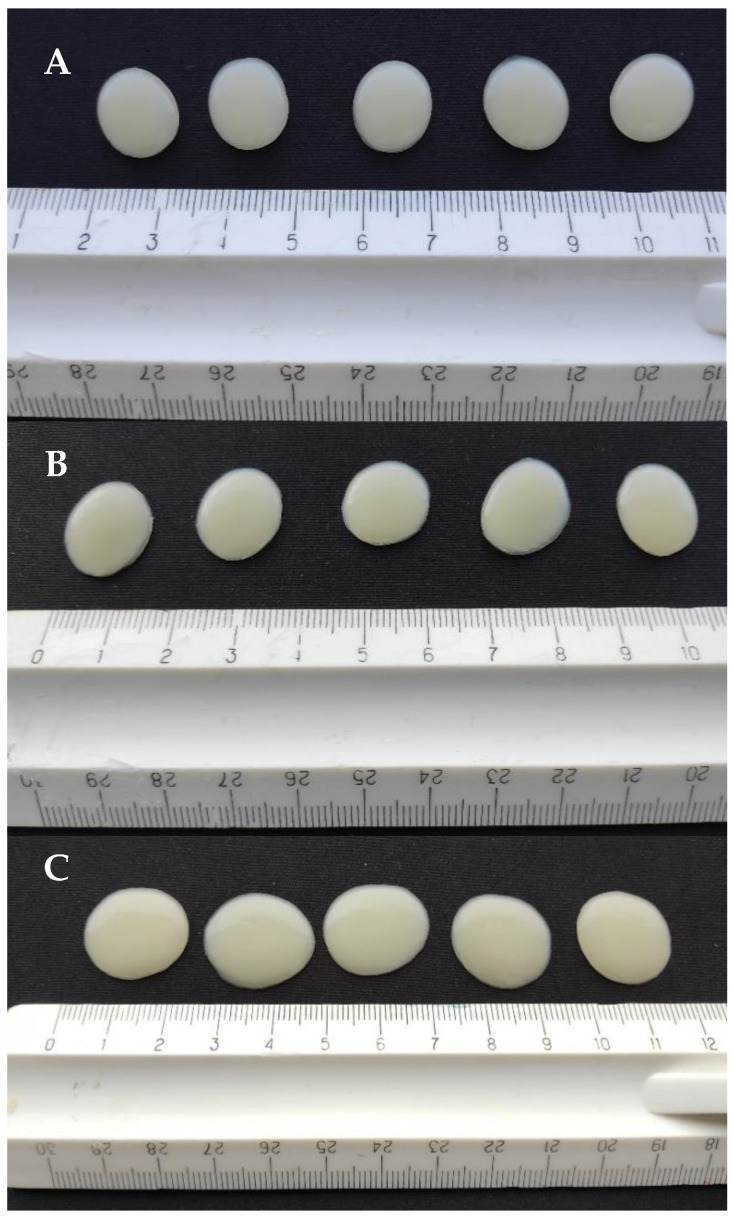
The gel tablets (*n* = 5) prepared using the automated semi-solid extrusion (SSE) material deposition method. Key: (**A**) Reference gel tablets (without any API); (**B**) Gel tablets with 3.0 mg of propranolol HCl; (**C**) Gel tablets with 5.0 mg of propranolol HCl.

The gel tablets prepared using the automated SSE material deposition method were mostly round or slightly oval in shape (Figure 6). The surface of the tablets was soft, elastic, and smooth. The printing mixture was evenly distributed, being slightly thinner at the edges and thicker in the middle. As expected, the gel tablets loaded with 3.0 mg of API (with a target tablet weight of 300.0 mg) presented the smallest diameter (size), while the gel tablets loaded with 5.0 mg of API (500.0 mg) presented the largest diameter and size (Figure 6 and Table 2). Since the content of propranolol HCl was very small (1%), no significant effects of API on the physical appearance and morphological characteristics of the gel tablets were observed.

**Figure 7:**
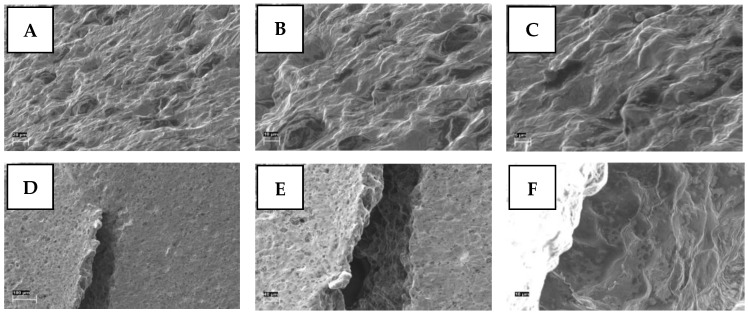
Scanning electron microscopy (SEM) images of the cross-section (**A**–**C**) and surface (with a potential crack or defect) (**D**–**F**) of the gel tablets prepared using the automated semi-solid extrusion (SSE) material deposition method, and loaded with 1% (*w*/*w*) propranolol HCl. Key: Magnification scale bar 20 µm, 10 µm, 5 µm (**A**–**C**), and 100 µm, 40 µm, 10 µm (**D**–**F**), respectively.

The SEM images taken from the cross-section and upper surface of the gel tablets loaded with 1% (*w*/*w*) API are shown in Figure 7. The tablets presented a porous internal structure as seen in the SEM images on the cross-section of the API-loaded gel tablet (Figure 7A–C). The surface of the gel tablet was relatively smooth, with tiny pores on it. However, we observed small cracks with a diameter of 100–200 μm on the surface of the tablets as surface defects (Figure 7D,F), thus suggesting the presence of internal stress and strain in these tablets. Figure 7E also reveals that the crack wall on the surface (i.e., the internal structure of the gel tablets) has a porous structure.

### 3.3. Weight and Weight Variation of Gel Tablets

The MiniLab control program of our automated SSE material deposition system was programmed to generate the gel tablets with an exact specified mass. The program was set for printing the gel tablets with a 1% (*w*/*w*) API content and for printing the desired number of the tablets. We used three different theoretical contents of API (3.0 mg, 4.0 mg, and 5.0 mg) corresponding to the (gel tablet) target masses of 300.0 mg, 400.0 mg, and 500.0 mg, respectively. The amount of mixture coming out of the printing syringe was regulated by pressure applied to the piston (non-changeable by printer user) and the range of movement of the piston, which was programmed based on the volume and expected viscosity of the mixture to be printed out.

The weights of the gel tablets (*n* = 25) were recorded on an analytical balance 30 min after printing. This was followed by measuring the diameter and height of the tablets (Table 2). The weight and diameter of the gel tablets prepared from the API-loaded (1% *w*/*w*) CuraBlend™ semi-solid mixtures were larger than those of the reference gel tablets printed from the CuraBlend™ mixture without API (propranolol HCl). The gel tablets were oval to round in shape, and the diameter of the tablets loaded with 3.0 mg, 4.0 mg, or 5.0 mg of API was 0.5 cm, 1.5 cm, and 2.0 cm, respectively (Figure 6 and Table 2). Interestingly, the gel tablets loaded with API exhibited a significantly larger diameter and smaller height compared to the reference gel tablets without API (Table 2). This is obviously due to the lower viscosity of the API-loaded printing mixtures (as described in Section 3.1), thus enhancing the spreading of the mixture on the printed bed.

The gel tablets were not blistered to MediCup immediately after printing. Since the humidity in the laboratory was relatively low, it is evident that the gel tablets may have dried (i.e., lost their moisture) and solidified after printing, causing their weight to decrease. In this study, we did not investigate the effects of storage in blister packaging on the weight of the tablets.

### 3.4. Physicochemical Changes in the API Loaded in the Gel Tablets

**Figure 8:**
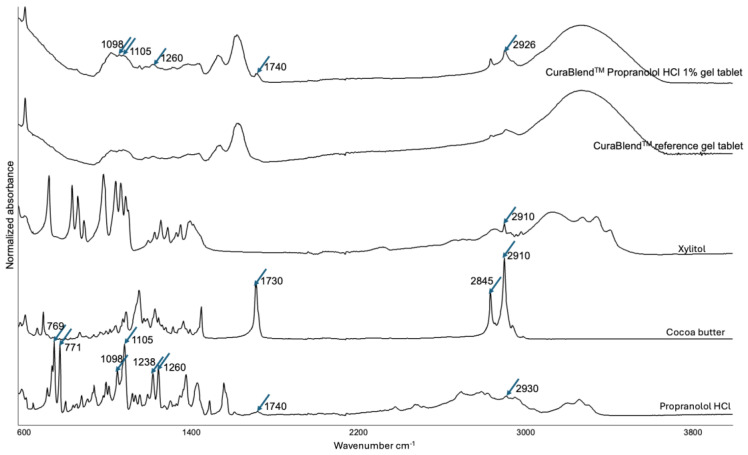
Fourier transform-infrared (FTIR) spectra for propranolol HCl in a powder form, cocoa butter, xylitol, the CuraBlend™ reference gel tablet without API (propranolol HCl), and the CuraBlend™ gel tablet loaded with 1% (*w*/*w*) API.

Since automated extrusion-based material deposition is a novel method for additive manufacturing, we were also interested in revealing potential process-induced transformations (PITs) in the formulations of propranolol hydrochloride used in this technology. Figure 8 shows the FTIR spectra for propranolol hydrochloride in powder form, cocoa butter, xylitol, and the reference gel tablets without API and with 1% (*w*/*w*) of API. As seen in Figure 8, the FTIR spectra of the reference gel tablet and the gel tablet loaded with 1% (*w*/*w*) API are quite similar. However, the FTIR spectrum of the gel tablet loaded with API (propranolol HCl) has five distinguishable peaks that coincide with the corresponding spectrum of propranolol hydrochloride powder at 1098 cm^−1^, 1105 cm^−1^, 1260 cm^−1^, 1740 cm^−1^ and 2926 cm^−1^ which are not present or presented in lower intensity in the FTIR spectrum of the reference gel tablet. The characteristic peaks within 2715–2960 cm^−1^ were caused by a secondary amine group, while the individual peak at approximately 1260 cm^−1^ was caused by aryl alkyl ether [19]. The results suggest the absence of PIT related to the use of propranolol HCl in this gel tablet formulation. It is evident that the low intensity of the two characteristic peaks for API (in the FTIR spectrum of the gel tablet) was due to the low concentration of API in these formulations that were intended for pediatric drug therapy.

**Figure 9:**
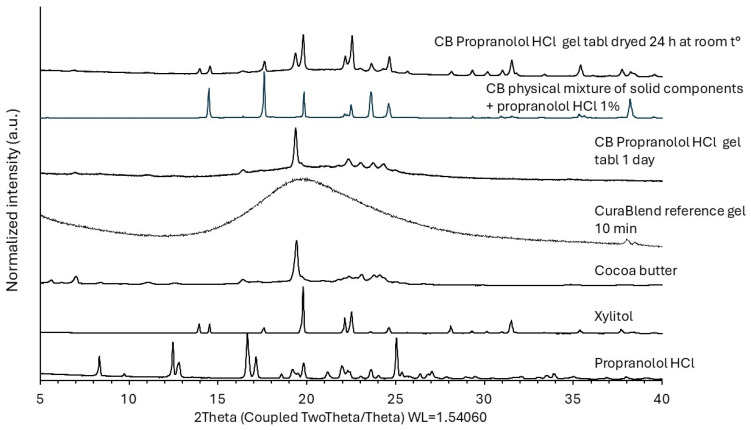
X-ray powder diffraction (XRPD) patterns (from the bottom to top): propranolol HCl and the two key excipients (xylitol and cocoa butter) of a CuraBlend™ gelatin-based gel, CuraBlend™ reference gel (without API), CuraBlend™ (CB) gel tablet loaded with API 1% (*w*/*w*) and stored in a blister package for one day, the physical mixture of CuraBlend™ solid components and propranolol HCl 1% (*w*/*w*), and the unpacked CuraBlend™ gel tablet loaded with API 1% (*w*/*w*) and dried at room temperature for 24 h.

Figure 9 presents the XRPD patterns of the pure materials (propranolol HCl and key excipients), CuraBlend™ reference gel (without API), the physical mixture of CuraBlend™ solid materials and propranolol HCl 1% (*w*/*w*), and the final gel tablets without or with API stored either in a blister package or unpacked for 24 h at room temperature. The XRPD pattern of the CuraBlend™ gel displayed an “amorphous halo” characteristic to the gelatin as the main excipient of a CuraBlend™ printing gel base. The XRPD diffractogram of propranolol HCl shows numerous characteristic sharp peaks due to its crystalline state, as has been seen in previous studies [20]. The XRPD pattern for the physical mixture of CuraBlend™ solid excipients and propranolol HCl 1% (*w*/*w*) showed the peaks characteristic of xylitol and cocoa butter, which are the key excipients in CuraBlend™ printing gel. The characteristic peaks for propranolol HCl, however, were not obviously detectable due to the low concentration of API. On the other hand, it is evident that water-soluble propranolol HCl exists in the form of a solution in the gel tablets, since the gel tablets contain quite a high amount of residual water. As seen in Figure 9, the characteristic peaks for water-soluble propranolol HCl are not distinguishable in the XRPD pattern of the gel tablets stored in a blister package, thus suggesting that the API exists in the form of a solution in these CuraBlend™ gel tablets. We have found that poorly water-soluble furosemide (unpublished data) remains in a crystalline form (XRPD) at the drug load of 1%, 2%, and 5% in the corresponding gel tablets stored in a blister package and does not dissolve in the residual water of gel tablets, unlike water-soluble propranolol HCl. Therefore, the present XRPD results reveal that propranolol HCl exists in the form of a solution due to residual water in the present gel tablets.

The XRPD analyses enable us also to describe the potential changes in the crystallinity of the two key excipients (xylitol and cocoa butter) in the gel tablets. To support this, the XRPD patterns were recorded not only for the CuraBlend™ gelatin-based printing gel and gel tablets, but also for the individual pure materials and physical mixture of solid materials of CuraBlend™ printing gel base in order to demonstrate the correspondence of characteristic peaks. Since CuraBlend™ printing gel base is a multi-component semisolid blend, there are also additional peaks in the XRPD diffractograms of the gel tablets. As seen in Figure 9, the XRPD patterns of gel tablets shows also the characteristic peaks for the two key excipients, xylitol and cocoa butter. Xylitol as a water-soluble excipient exists in the form of a solution (like propranolol HCl) in the CuraBlend™ gel tablet loaded with API 1% (*w*/*w*) and stored in a blister package for one day. This is due to the high level of residual water entrapped in the gelatin-based gel tablets. However, the XRPD pattern for the corresponding unpacked CuraBlend™ gel tablets dried at room temperature, reveals that xylitol re-crystallizes within 24 h in such gel tablets due to the partial evaporation of water (Figure 9).

According to the literature, cocoa butter presents complex crystallization behavior and has a total of six known crystal polymorphic forms [21,22,23]. While the gel tablets were solidifying, it is evident that cocoa butter transferred to its polymorphic form(s), similar to what has been reported in other substances, e.g., chocolate [22,23]. With the present gel tablets, we observed that cocoa butter exists in its β(V) crystal polymorphic form (Figure 9).

### 3.5. Mechanical Properties

**Figure 10:**
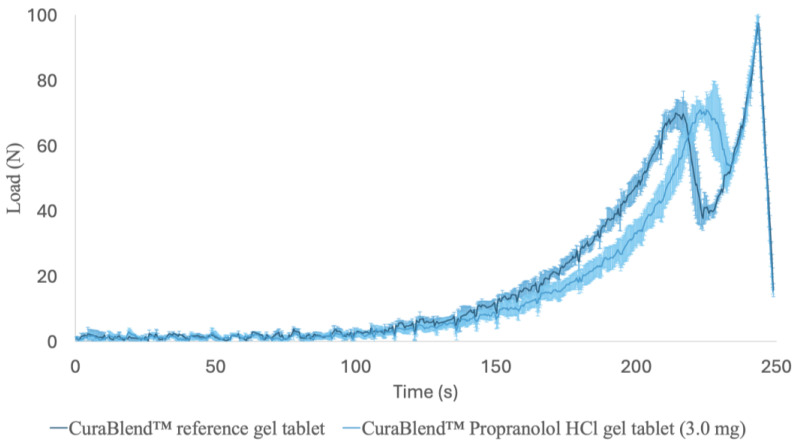
The characteristic force-time profiles in a uniaxial tablet compression test for the printed CuraBlend™ reference gel tablets (without API) (*n* = 6) and the printed CuraBlend™-propranolol HCl (3.0 mg) gel tablets (*n* = 6). The mechanical strength values were measured with a Lloyd LRX material tester using a constant speed (1.000 mm/min) for the crosshead.

The printed gel tablets were elastic, soft, and jelly-like chewable drug preparations. The characteristic compression force-time profiles for the CuraBlend™ reference gel tablets (without API) and the propranalol HCl-loaded (3.0 mg) CuraBlend™ gel tablets are shown in Figure 10. These curves show the resistance of the gel tablet under a constantly increasing load (N) as a function of time (min). The maximum load needed for the final deformation (“breaking down”) of the CuraBlend™ reference gel tablets was very uniform, ranging from 65 N to 75 N. The maximum load needed for the final deformation of the propranolol HCl-loaded (3.0 mg) CuraBlend™ gel tablets was even more uniform and slightly higher, ranging from 73 N to 80 N. The time for the maximum load at break (Tmax) was different between the reference and API-loaded gel tablets (3.5 min vs. 3.7 min).

### 3.6. Dissolution In Vitro

**Figure 11:**
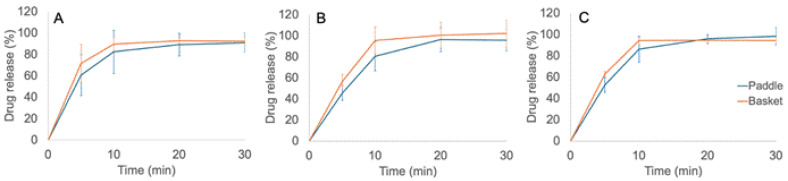
In vitro drug release curves for the printed CuraBlend™-propranolol HCl gel tablets prepared using the automated extrusion-based material deposition method (*n* = 6). The printed gel tablets contained (**A**) 3.0 mg, (**B**) 4.0 mg, and (**C**) 5.0 mg of propranolol HCl. The dissolution tests were performed using both the basket (orange curve) and paddle (blue curve) methods.

As seen in Figure 11, more than 85% of the drug load was released from the printed gel tablets within 10–15 min, thus confirming the immediate-release dissolution of these drug preparations. According to the United States Pharmacopoeia (USP 35), no less than 75% of the API load should be released within 30 min at a rotation speed of 100 rpm, in order to comply with the in vitro dissolution requirements of oral immediate-release tablets. As seen in Figure 11, the drug release was 100% within 30 min with these printed gel tablets. Therefore, the dissolution of the gel tablets prepared using the automated SSE material deposition method complied with the corresponding requirements of USP 35. As seen in Figure 11, we did not find any significant differences in the dissolution profiles of the printed gel tablets between the basket and paddle methods. The in vitro dissolution of API (propranolol HCl) was slightly faster when the tablets were tested using the basket method compared to the paddle method. This is obviously due to the slight differences in the sampling point, mixing pattern, and capacity between basket and paddle dissolution methods. In the basket method, the sample is taken closer to the printed gel tablet in the dissolution vessel than it is in the paddle method. Moreover, it is evident that rotating baskets do not stir the dissolution medium as intensively as rotating paddles. Consequently, the concentration of API is initially higher in the closed vicinity of the baskets.

## 4. Conclusions

Automated extrusion-based material deposition is a feasible method for preparing customized chewable gel tablets intended for pediatric drug therapy. The method enables elastic and small-sized gel tablets to be prepared based on a CuraBlend^TM^ polymeric mixture and loaded with 1% (*w*/*w*) propranolol HCl. The printing performance of the method, however, is dependent on the temperature and viscosity of the drug-loaded gel. The viscosity of the semi-solid CuraBlend^TM^ polymeric mixture is dependent on the concentration of propranolol HCl, which in turn affects the printing performance of the gel tablets. The inclusion of propranolol HCl in CuraBlend^TM^ decreases the viscosity of the mixture, but this does not significantly impair the printing performance of the semisolid mixture. The small weight variation among the printed gel tablets (less than 5%) suggests the uniform and stable performance of the formulation using the automated extrusion-based material deposition method. Water-soluble propranolol HCl exists in the form of a solution in the gel tablets due to a high amount of entrapped water in such gelatin-based tablets. The inclusion of propranolol HCl in the CuraBlend^TM^ gel base slightly enhances the mechanical properties of the printed gel tablets. The dissolution test in vitro verifies that the printed gel tablets are immediate-release drug preparations. More research is needed to identify the key material and process parameters using the automated extrusion-based material deposition method and to reveal the effects of these parameters on the performance and stability of gel tablets.

## Figures and Tables

**Table 1:** Comparison of the dissolution test method described in the United States Pharmacopoiea (USP 35) for Propranolol Hydrochloride tablets and the modified in-house dissolution test method used for these gel tablets.

Parameter	USP 35	Modified (In-House)
Apparatus	Basket	Paddle and basket
Rotation speed	100 rpm	100 rpm
Dissolution medium	0.063 M HCl, 900 mL	0.063 M HCl, 900 mL
Temperature	36.7 ± 0.2 °C	36.7 ± 0.2 °C
Time	60 min	45 min
Analytical wavelength (HPLC)	292 nm	292 nm

**Table 2:** Average weight, diameter, and height of gel tablets prepared using the automated semi-solid extrusion (SSE) material deposition method and loaded with propranolol HCl at a theoretical content of 3.0 mg, 4.0 mg, and 5.0 mg (*n* = 25).

Response	Gel Tablet Prepared Using the Automated SSE Material Deposition Method					
	Tablet Strength 3.0 mg (Mean ± SD)		Tablet Strength 4.0 mg (Mean ± SD)		Tablet Strength 5.0 mg (Mean ± SD)	
	Ref. Gel Tablet (Without API)	CuraBlend™ Gel Tablet Loaded with API (1%)	Ref.	CuraBlend™ Gel Tablet Loaded with API (1%)	Ref.	CuraBlend™ gel Tablet Loaded with API (1%)
Weight (mg)	280 ± 8.0	282 ± 8.3	375 ±10.6	378 ± 7.6	468 ±13.1	481 ± 20.8
Diameter (mm)	12.7 ± 0.2	14.8 ± 0.7	14.7 ± 0.3	17.1 ± 0.8	16.5 ± 0.3	20.2 ± 1.0
Height (mm)	2.7 ± 0.1	2.1 ± 0.2	2.7 ± 0.1	2.1 ± 0.1	2.7 ± 0.1	2.0 ± 0.1

## Data Availability

The original contributions presented in the study are included in the article. Further inquiries can be directed to the corresponding author.
